# The local ordering of polar solvents around crystalline carbon nitride nanosheets in solution

**DOI:** 10.1098/rsta.2022.0337

**Published:** 2023-10-30

**Authors:** Martin C. Wilding, Chris Benmore, Thomas F. Headen, Camilla Di Mino, Thomas S. Miller, Theo M. Suter, Furio Corà, Adam J. Clancy, Andrea Sella, Paul McMillan, Christopher A. Howard

**Affiliations:** ^1^ UK Catalysis Hub, Research Complex at Harwell, Rutherford Appleton Laboratory, Oxfordshire OX11 0QX, UK; ^2^ ISIS Neutron and Muon Source, Science and Technology Facilities Council, Rutherford Appleton Laboratory, Oxfordshire OX11 0QX, UK; ^3^ X-Ray Science Division, Advanced Photon Source, Argonne National Laboratory, Argonne, IL, USA; ^4^ Department of Physics and Astronomy, University College London, London WC1E 6BT, UK; ^5^ Department of Chemistry, University College London, London WC1E 6BT, UK; ^6^ Electrochemical Innovation Laboratory, Department of Chemical Engineering, University College London, London WC1E 7JE, UK

**Keywords:** neutron diffraction, high-energy X-ray diffraction, carbon nitride, liquid structure, solvent ordering

## Abstract

The crystalline graphitic carbon nitride, poly-triazine imide (PTI) is highly unusual among layered materials since it is spontaneously soluble in aprotic, polar solvents including dimethylformamide (DMF). The PTI material consists of layers of carbon nitride intercalated with LiBr. When dissolved, the resulting solutions consist of dissolved, luminescent single to multilayer nanosheets of around 60–125 nm in diameter and Li+ and Br− ions originating from the intercalating salt. To understand this unique solubility, the structure of these solutions has been investigated by high-energy X-ray and neutron diffraction. Although the diffraction patterns are dominated by inter-solvent correlations there are clear differences between the X-ray diffraction data of the PTI solution and the solvent in the 4–6 Å^−1^ range, with real space differences persisting to at least 10 Å. Structural modelling using both neutron and X-ray datasets as a constraint reveal the formation of distinct, dense solvation shells surrounding the nanoparticles with a layer of Br^−^close to the PTI-solvent interface. This solvent ordering provides a configuration that is energetically favourable underpinning thermodynamically driven PTI dissolution.

This article is part of the theme issue 'Exploring the length scales, timescales and chemistry of challenging materials (Part 2)'.

## 1. Introduction

The manipulation of nanoparticles in liquids is important when considering their scalable implementation in a range of technologically important applications. For example, liquids with de-agglomerated nanoparticles can be used to print, assemble or embed active nanoparticles into coatings, films, composites and functional materials [1,2]. In this contribution, we present the results of a study of the structure of such a liquid containing carbon nitride nanoparticles. The exceptional chemical and thermal stability of polymeric carbon nitrides and their numerous technical applications has made them the subject of much recent study [3–5]. Since the isolation of graphene sheets and recognition of the potential application of semi-metallic graphene in electronic devices [6], there has been interest in other two-dimensional, earth abundant materials which are more scalable [5,7,8]. Polymeric carbon nitride (ideally of stoichiometry C_3_N_4_) is one such class of semiconducting materials [5,8,9], with applications that include photocatalysis and this has, in turn, led to interest in the related carbon nitride materials that exhibit similar properties. Although the synthesis can be complex [10–13], poly-triazine imide (PTI) has emerged as one such candidate, and it can be produced in bulk by reacting organic precursors with molten salt mixtures [13–16]. One of the many interesting properties of PTI is its ability to dissolve spontaneously in aprotic, polar solvents and these dispersions or solutions [2] have many technical applications [17–22].

**Figure 1.**
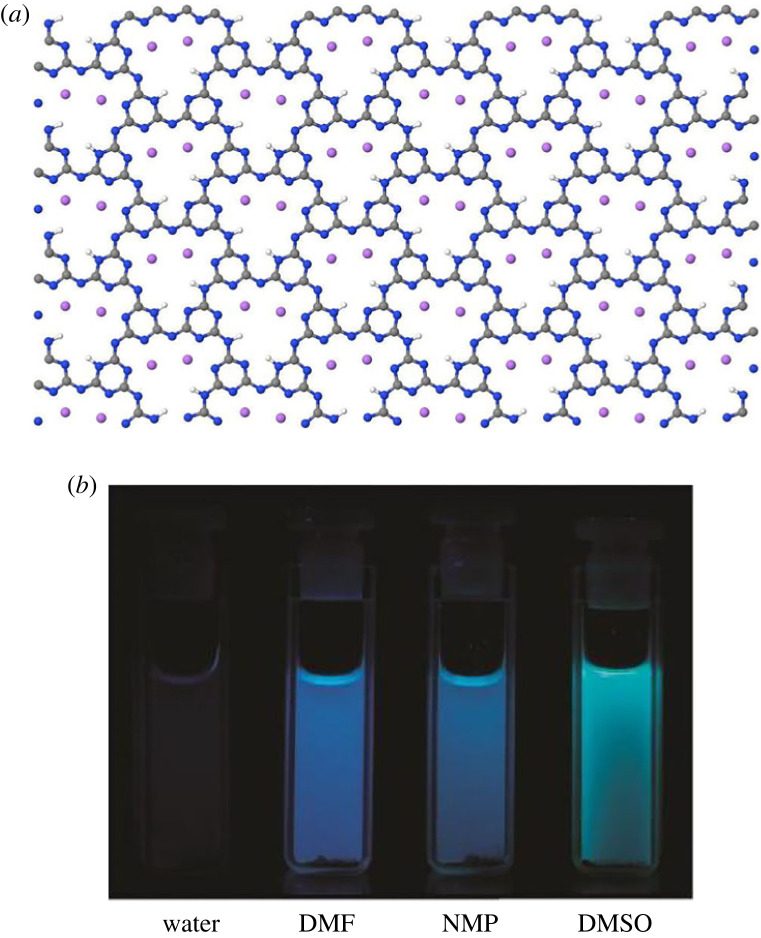
The structure of a poly-triazine imide (PTI) sheet showing the large pores formed by linking triazines by NH bridges and the location within these pores of the Li+ resulting from the LiBr or LiCl synthesis. Bromine or chlorine ions are located between the carbon nitride sheets (*a*). PTI can spontaneously dissolve into aprotic polar solvents including DMF and DMSO (*b*) showing characteristic UV-visible luminescence.

Poly-triazine imide (PTI) is a highly crystalline intercalated graphitic carbon nitride produced by the reactive polymerization of dicyandiamide in a eutectic molten salt (KCl–LiCl or KBr–LiBr) [13–16]. The resulting structure is one of 1,3,5 triazine rings linked by N–H bridges and forms a slightly bucked two-dimensional sheet with a C_6_N_9_H_3_ unit cell and voids within the crystal structure (figure 1*a*). The PTI layers are intercalated with the lithium and halide components from the molten salt bath. While the halide ions occupy the voids within or between the planar sheets, lithium both partly replaces some of the hydrogen in the amide bridges while an excess balances the halide change. The intercalated salt can be removed, e.g. by Soxhlet extraction and dried, which exchanges Li+ with H+ forming pure, intercalant-free PTI. The intercalant-free PTI can undergo re-intercalation treatment with an ionic solvent [19] or intercalated with water molecules from the atmosphere, whereupon the water molecules have been shown to move through the large channels formed by aligned, planar layers [21].

There is substantial interest in exfoliating gCN materials in liquids to create metastable suspensions that enable their scalable manipulation for applications [21]. However, the formation of suspensions from typical gCN materials of polymeric is limited because of the high degree of cross-linking. To overcome these barriers, high shear stress, induced by sonication, is used to produce kinetically stable suspensions that are thermodynamically metastable. In contrast, intercalated PTI materials can form thermodynamically stable solutions that spontaneously form on direct contact with aprotic (no hydrogen exchange), polar solvents such as *N′N*-dimethylformamide (DMF), dimethyl sulfoxide (DMSO), *N,N* dimethylacetamide (DMAC) and *N*-methyl-2-pyrrolidone (NMP), without damage to the PTI structure. Exfoliation has been demonstrated and is evidenced by a change in colour and also by the characteristic UV-visible luminescence (figure 1*b*) [2,21]. The solutions comprise hexagonal nanosheets which are between 30 and 165 nm in lateral dimensions, and Ih are between 2 and 25 layers thick (although mostly these comprise nine triazine layers, therefore the average thickness of the nanosheets is 2–3 nm). The presence of the nanosheets is indicated by the photoluminescence of the solutions. While several solvents result in this thermodynamically driven dissolution of the intercalated PTI, the highest concentration of dissolved or dispersed nanosheets occurs in DMSO [21,23]. There is co-dissolution of the intercalating species with the PTI, Li^+^ and Br^−^ or Cl^−^ ions also enter the solution, although the solutions are not saturated with chloride or bromide. Although the highest concentration of PTI occurs in DMSO this solvent is very hygroscopic, and the presence of H_2_O dramatically alters the dissolution of PTI [21].

Nanoparticle dispersions in liquids are traditionally viewed as metastable suspensions, where the charge on the surface of the colloid is an important parameter. DVLO (Derjaguin–Landau–Verwey–Overbeek) theory is the most common method used in describing colloidal suspensions and is effective at describing relatively large, dispersed particles. However, as discussed by Batista [22] as the size of nanoparticles becomes comparable to the size of solvation shells, characteristics of the dispersion can deviate from that anticipated from classical colloidal models [1,2]. In a classic colloidal suspension, there is a balance of repulsive and attractive forces between dispersed species and the solvent is treated as a uniform density dielectric continuum. However, as the particle size of the dispersed nanoparticle approaches that of the solvent layer, a range of different behaviours is found that differs to those predicted for colloidal models, and the local solvent ordering can play a significant or even dominant role [2,23]. Solvent can potentially balance steric, charge-screening and hydrogen bonding and provide the energetic driving force necessary for spontaneous dissolution, for example in simple ionic salts in water, in which the solvent-coordinated solute system is energetically favourable compared to the combination of undissolved salt and pure solvent. The solvation shell structures around nanoparticles have been shown to persist to distances of up to 15 Å [24] and suggest that the final dissolved product is intermediate between a simple solution and a colloidal suspension. The dissolution of gCN materials such as PTI in aprotic polar solvents is not well understood, and although polar solvents are required to dissolve these gCN materials, not all polar solvents are equally successful suggesting that the dissolution mechanism may be underpinned by solute-driven restructuring of the solvent [23].

The re-organization of solvent molecules around nanoparticles has been studied extensively by Zobel *et al.* [24,25]. The influence on bulk liquid structure can potentially be evaluated by comparing the bulk liquid structure, obtained from total neutron or X-ray scattering, with that of a liquid containing the nanoparticle and the resulting difference in density, seen as a decaying, sinusoidal oscillation with possible additional contributions from the nanoparticle is obtained if there is a shell structure surrounding the nanoparticle. This comparison of total scattering patterns has been used, for example, to provide direct evidence for the interaction of iron oxide nanoparticles with their surrounding solvent [24] with high-energy X-ray scattering of these colloidal dispersions used to identify solvation shells. X-rays are only weakly sensitive to hydrogen, the most abundant atom in many polar solvents such as DMF, and neutron scattering can, therefore, be used to evaluate the differences in structure of gCN solutions and the bulk H-rich solvent. Given the importance of H interactions in solvent ordering around any solute, neutrons are uniquely powerful for experimentally investigating this arrangement [23]. Importantly, the scattering lengths of H and D differ in sign and so by using isotopically distinct solvents the changes in the partial contribution of hydrogen to the total liquid structure can be discerned.

In this study, we use both neutron and X-ray total scattering to explore the changes in liquid structure as the PTI materials are dissolved. Solutions of PTI in the polar solvent, DMF were studied using high-energy (greater than 100 keV) X-ray scattering available at the Advanced Photon Source while neutron scattering with isotopic substitution was performed at the ISIS Neutron and Muon source using the NIMROD instrument. We present neutron data for PTI dissolved in DMF and a DMF liquid saturated with LiBr. On dissolution the PTI dissolution will release the intercalated lithium halide salts into the solvent and the structural changes could simply reflect the development of solvation shells around the Li^+^ and Br^−^ ions. In order, therefore, to understand the distinct dissolution mechanism of the PTI we compare the structure of the PTI–DMF solution with a PTI-free solution of DMF saturated with LiBr. Empirical potential structural refinement (EPSR) [26] is used to evaluate these structural changes, with the total scattering data used to constrain the EPSR models and reveal the role of solvent restructuring at the nanoscale.

## 2. Experimental set-up

### (a) Sample synthesis

Intercalated PTI was prepared from dicyanamide in a molten, eutectic LiBr/KBr or LiCl/KCl molten salt mixtures [12,13]. The lithium (Merck, LiBr and LiCl) and potassium salts (KBr and KCl) were mixed with 2 g of 99% dicyanamide (Merk) in a N_2_-filled glovebox and pre-treated under vacuum at 400°C before grinding and sealing under vacuum in a quartz ampoule. The ampule was then heated at 600°C for 16 h before washing with deionized water to remove excess salt. The formation of PTI intercalated with either LiCl (PTI–LiCl) or LiBr (PTI–LiBr) was confirmed by X-ray diffraction [19]. The solutions were prepared by placing at the bottom of a vial and adding DMF dropwise until the vial was full. After a period of several days, a layer of powder was still visible at the bottom of the vial but formation of the PTI solution was confirmed by colour change and by using a UV light to demonstrate luminescence [2].

### (b) High-energy X-ray diffraction measurements

High-energy X-ray diffraction (HEXRD) measurements were conducted at the high-energy beamline 6IDD at the Advanced Photon Source (APS), Argonne National Laboratory, using a monochromatic beam of 100.332 keV, (*λ* = 0.123574 Å). Liquid samples were loaded into low-background quartz glass capillaries mounted in transmission geometry with the incident beam collimated to 0.5 × 0.5 mm, the scattered X-rays are detected on a vertically mounted amorphous silicon detector with 2048 × 2048 pixels. Scattering patterns for the capillary and for liquids were collected on this two-dimensional detector and reduced to one-dimensional patterns by integrating all the pixels, using Fit2D [27] which corrects for polarization effects and geometry. Sample-detector distance and detector tilt corrections were obtained by calibration with a crystalline CeO_2_ standard. The resulting one-dimensional patterns were further processed using PDFgetX2 software [28] to obtain *S(Q)*, where *Q* scattering vector; and *g(r)* functions, where *g(r)* is the pair distribution function normalized to single atom scattering.

### (c) Neutron diffraction measurements

Neutron diffraction experiments were performed using the Near and InterMediate Range Order Diffractometer (NIMROD) instrument at the ISIS spallation neutron source (RAL, STFC, UK) [29]. This purpose-built diffractometer is optimized for structural studies of hydrogen-containing disordered materials and therefore uniquely suited to the study of PTI solutions in DMF. Data were collected for three sets of liquids with three different isotopic compositions each based on non-deuterated DMF, fully deuterated DMF, and a 1 : 1 molar mixture of the two monoisotopic solvents. Three liquids comprising the neat DMF solvent were compared with PTI solutions and with DMF liquids saturated with LiBr. Samples were loaded into flat-plate null-coherent scattering Ti_0.68_Zr_0.32_ alloy cells which have internal dimensions 1 mm × 35 mm × 35 mm and neutron diffraction data were collected between 0.02 Å^−1^ < *Q* < 50 Å^−1^. Sample cells were loaded onto the automatic room temperature sample changer and cycled into the 30 × 30 mm sample beam for a minimum of four hours. Scattering from the empty instrument and with empty sample cells were also collected. The measured neutron scattering was reduced to the interference differential scattering cross-section, *F(Q)*, using the GudrunN program [30] which merges the time-of-flight scattering from all detectors to a single *Q* scale and normalizes to an absolute scale by comparison with the scattering from a 3 mm vanadium-niobium plate. This programme also subtracts scattering from the sample container and empty instrument and applies corrections for beam attenuation and multiple scattering. Particular attention was paid to correction of inelasticity effects, especially for the samples containing hydrogen. The self-scattering background and inelasticity effects were removed from the total differential scattering cross-section using an iterative method developed by Soper [31].

### (d) EPSR modelling

Empirical Potential Structural Refinement (EPSR) [26] aims to maximize the information that can be extracted from a set of scattering experiments on a disordered system by iteratively refining a three-dimensional ensemble of molecules via tuning interatomic potentials, until the ensemble is consistent with the measured diffraction data. The method starts with an equilibrated Monte Carlo simulation based on initial ‘seed’ potentials. The technique allows known prior information, such as molecular geometry, overlap and electrostatic constraints to be built into the refinement procedure. The EPSR simulations discussed here include a simulation of the liquid DMF which is compared with the recent study of both DMF and DMAc [32]. An equilibrated Monte Carlo ensemble of the system was used as a starting point, seeded by pairwise (Lennard–Jones and Coulomb) potentials discussed by Vasudevan & Murshif [33] (table 1). Two inter-torsional angles (dihedrals) were defined within the DMF molecule to describe its skeletal planarity, as suggested by previous findings in the literature [34]. The methyl groups attached to the nitrogen atom were allowed to freely rotate around the C–N axis. The standard minimum image convention was applied to the ensemble with appropriate periodic boundary conditions. The experimentally derived atomic number densities for DMF liquids are 0.09330 Å^−3^ at 298 K. In order to model the solvent ordering, a surface of PTI was created using a single PTI layer (figure 1*a*) extended in the *a-* and *b-*crystallographic direction to form one side of the simulation box (58 × 34 Å), the dimension normal to the PTI layer forming the remainder of the box was 100 Å and this was filled with DMF molecules and 64 bromide ions to balance Li^+^, that is assumed to remain in the PTI, to mimic the solvent-nanoparticle interface. The modelled liquid structure is compared with the neutron and X-ray datasets and although the difference between modelled and experimental *F*(*Q*) will vary between each dataset, each dataset is given equal weighting.

**Table 1.** Lennard–Jones parameters and partial charges for the DMF molecule used in EPSR.

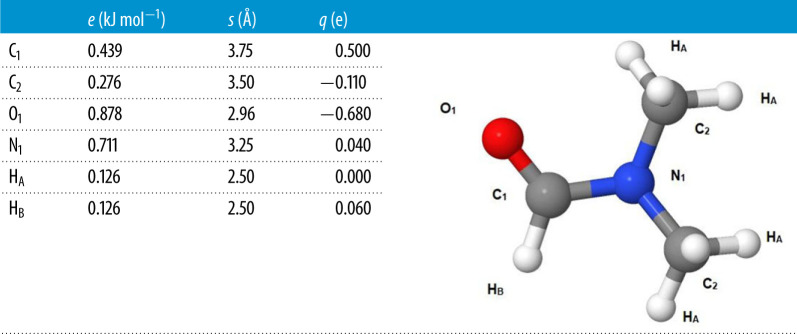

## 3. Results

### (a) High-energy X-ray diffraction

**Figure 2.**
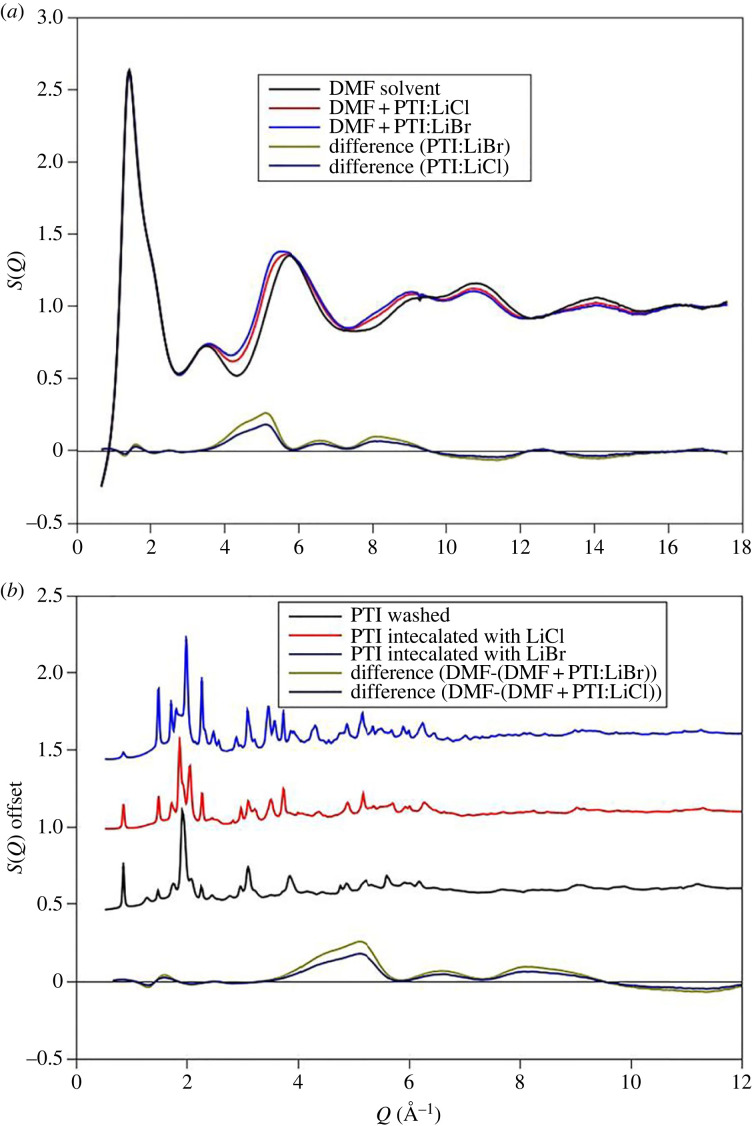
(*a*) High-energy X-ray diffraction data for DMF and DMF + PTI solutions with different intercalants. The residua for both the PTI intercalated with LiBr and LiCL are also shown. The main difference in in the 4–6 Å^−1^ range. (*b*) The difference in scattering patterns compared with crystalline PTI, with LiBr and LiCl and intercalant-free. The Bragg features for the crystalline material do not coincide with the features in the difference curves and the HEXRD pattern for the liquids does not represent the presence of crystalline material.

The total structure factors (*S(Q)*) for DMF and DMF + PTI liquids are shown in figure 2. The two PTI solutions were made from PTI intercalated with LiCl and LiBr. Data are available to a relatively high value of scattering vector and indicate a variation in liquid structure over a range of length scales. The *S(Q)* data corrections assume minor amounts of Li^+^ and Br^−^ or Cl^−^ present in the solution, however, the contributions of these components to the total scattering levels are small and do not indicate saturation in the intercalating species. There are minor differences in the intensity of the first peak in the diffraction data although the most obvious differences between the PTI solutions and the DMF solvent are in the approximately 4–5 Å^−1^ range. This would suggest changes in *short-range* ordering rather than the anticipated changes at lower Q expected from inter-molecular changes in liquid structure. The differences in the PTI solutions and DMF solvent are similar for both LiCl and LiBr intercalated PTI and although the differences are more pronounced for the PTI:LiBr samples the changes in liquid structure are the same for both solutions. The minor differences between the Cl^−^ and Br^−^ liquids most likely reflect the differences in X-ray form factors for Br and Cl with a greater partial contribution to the total structure factor from the higher-Z Br. However, the amount of the intercalating halide in these liquids is small and the scattering from both solvent and PTI solutions is dominated by scatter from carbon and nitrogen atoms. The X-ray scattering from lithium is so weak that the contributions to the total scattering are negligible (table 2). A comparison with the *S(Q)* obtained for crystalline PTI and the PTI solutions shows that the positions of the Bragg peaks from the intercalated and intercalant-free [19] gCN materials do not match the peaks seen in the differences between the DMF liquid and the PTI solutions. We do not see a change in total scattering that can be attributed to PTI contributions and assume this is because it is too dilute, we suggest therefore that the differences in the scattering pattern between the DMF solvent and the PTI solution result from solvent–solvent and solvent–nanosheet interactions.

**Table 2.** Faber–Ziman weightings for X-ray and neutron partial structure factors for DMF and saturated LiBr–DMF solutions. These are shown as total absolute scattering, the contribution to the total structure factor (in %).

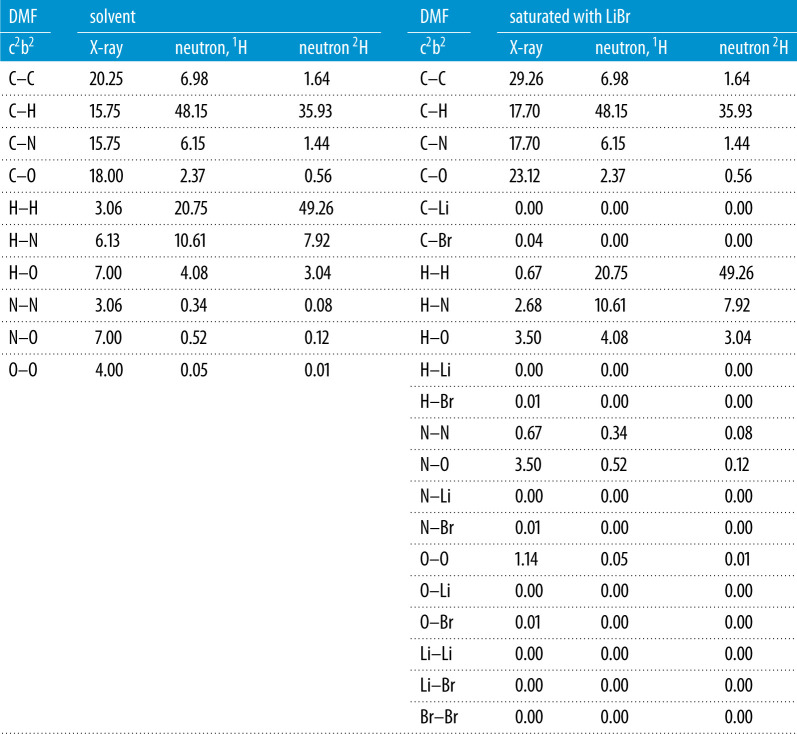

**Figure 3.**
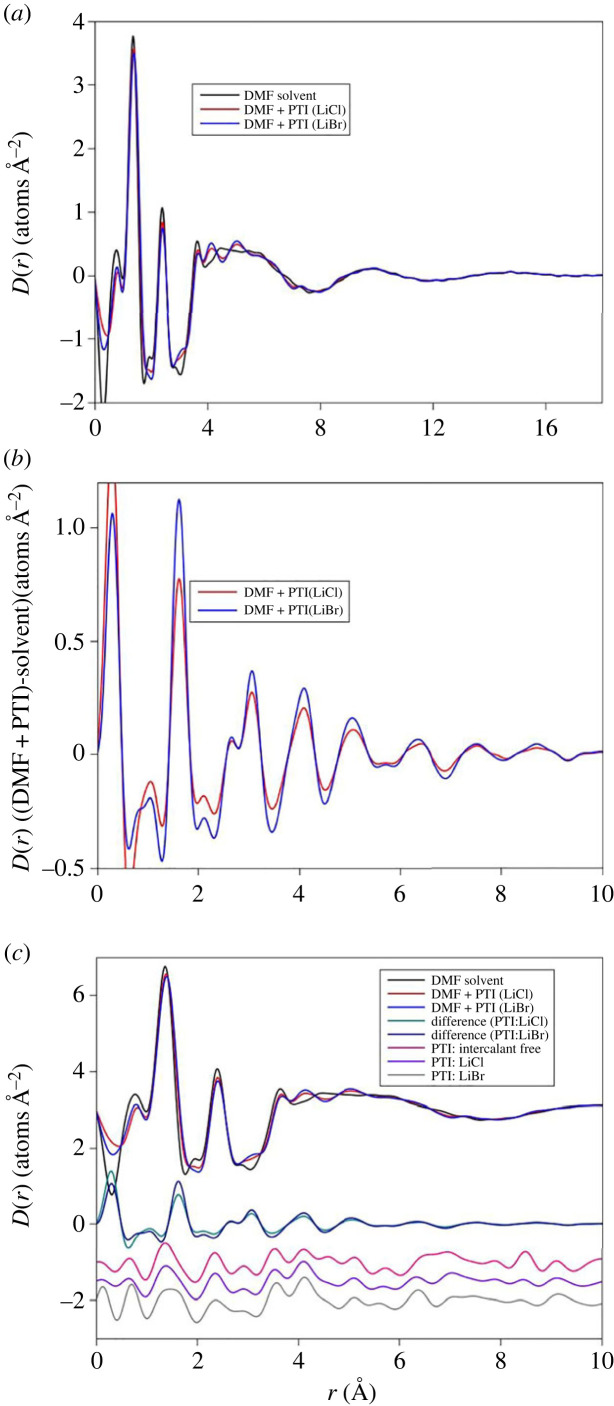
Transforms to real space of the high-energy X-ray diffraction data shown in figure 1, presented as *D*(*r*). These too are for DMF and DMF + PTI solutions with different intercalants. Note that this demonstrates substantial reordering of the solvent in the 3–6 Å range (*a*). To illustrate these differences the transform is only shown between 0 and 12 Å (*b*). The difference between the solvent and the PTI solutions show density waves then persist to distances of at least 12A. The *D*(*r*) for the crystalline PTI materials is shown (*c*), scaled to 20%, this is compared with the difference in liquid *D*(*r*) data and the liquid data. This illustrates the barely discernible contribution of crystalline PTI to the liquid scattering patterns and also that the differences in liquid structure (plotted on the same scale) occurs at radial distances that are different from the short-range ordering of the DMF molecule (i.e. it is broadening) and is also different from the C–N correlations in PTI.

The real space differences are shown in figure 3. Comparing the results from the PTI solutions with the DMF liquid shows a decrease in intensity of the first peak in the pair distribution function (shown as *D(r)* = 4*πρrg*(*r*)) at 1.36 Å, this peak comprises overlapping C–O and C–N correlations from the DMF molecule, and the difference reflects the asymmetric broadening of this peak (shift to higher radial distance). In figure 3*c*, the differences in real space are compared with the *D(r)* obtained for the PTI materials, the peak positions for the PTI overlap at low radial distance, however, the intensity of the first two peaks in the PTI solutions is reduced relative to the DMF solvent not increased and the differences at higher radial distance do not match the PTI peaks. There are differences in the peak at 2.4 Å, but the most obvious change in the pair distribution function with the formation of peaks at 4.1 Å and 5.0 Å in both PTI:LiBr and PTI:LiCl solutions, peaks that are absent in DMF. The differences between the solvent and the PTI solution pair distribution functions, plotted as Δ*D*(*r*), persist to at least 10 Å. These damped, sinusoidal oscillations occur in solutions with both LiCl and LiBr although they are more prominent in the Br-bearing liquid. The peak positions are identical in both PTI solutions. As with the reciprocal space data, it is possible that there are contributions from the crystalline PTI nanoparticles and in figure 3*c* the *D*(*r*) for intercalated and intercalant-free PTI are plotted, together with the difference between the PTI solutions and DMF liquid and the *D(r)* for the three liquids. The peak positions for the gCN do not coincide with the differences in liquid pattern and indicate that even if the concentration of PTI far exceeded that observed for PTI in DMF (approx. 0.8 mg ml^−1^ [2] and as high as 32 mg ml^−1^ for PTI in DMSO [21]) and would not account for the difference in the scattering patterns of the PTI solutions when compared to the DMF solvent. We concluded therefore that these changes represent a restructuring of the solvent around the PTI nanosheet when dissolved, the scattering is dominated by contributions from carbon atom pairs, as discussed above (table 2) and these changes in structure are unlikely to result from Cl^−^ or Br^−^ partial contributions to the total scattering. Similarly, the differences in liquid structure do not reflect a contribution from the C–C or C–N bond distances in the gCN to the scattering pattern.

### (b) Neutron diffraction

**Figure 4.**
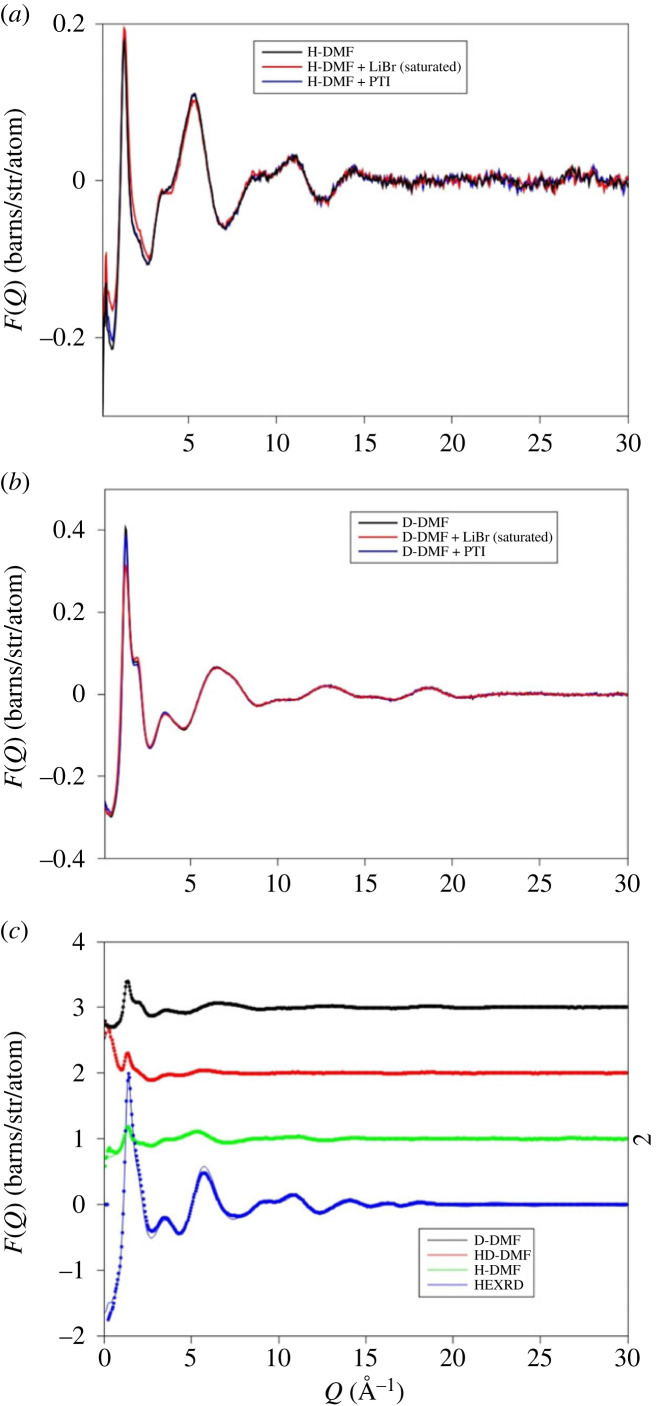
Results of neutron scattering experiments with isotopic substitution. The merged interference patterns for the D- and H-liquids are shown in parts *a* and *b*. These show minor differences between the solvent (DMF) and the PTI solutions. Also shown are the liquid patterns for LiBr-saturated DMF, the differences in structure between this latter liquid and DMF is more obvious. The results of EPSR fits to DMF which include the HEXRD data are shown in part *c*, this uses the potentials discussed in the text and provided in table 1.

The neutron scattering experiments were performed on DMF solutions prepared separately from those used for the HEXRD although the samples were prepared in the same way and show the same characteristic luminescence [2]. The HEXRD study showed the greatest differences are between DMF and a solution made with PTI intercalated with LiBr. A comparison of the Δ*D*(*r*) obtained from HEXRD indicates that the contributions from crystalline PTI nanoparticles are small, especially at such low concentration (figure 3*c*). In addition, the weighting of each partial contribution to total scattering (table 2) confirms that the differences in scattering between PTI solutions and the DMF solvent do not represent scattering contribution from bromine. However, it is possible that the differences in structure of the PTI solutions and liquid DMF reflect a change in solvent structure as the PTI is exfoliated and LiBr enters the solution. For the neutron scattering experiments therefore DMF saturated with LiBr, a solution produced from PTI intercalated with LiBr and the DMF solvent were all studied, each with three isotopic compositions (H-DMF, D-DMF and HD-DMF) (figure 4). The DMF solvent is compared with the study of Basma [32] using the EPSR seed potentials outlined in table 1 and to evaluate the HEXRD *S(Q)* for the DMF solvent. Figure 4*a* shows the scattering patterns and EPSR fits for the DMF solvent.

**Figure 5.**
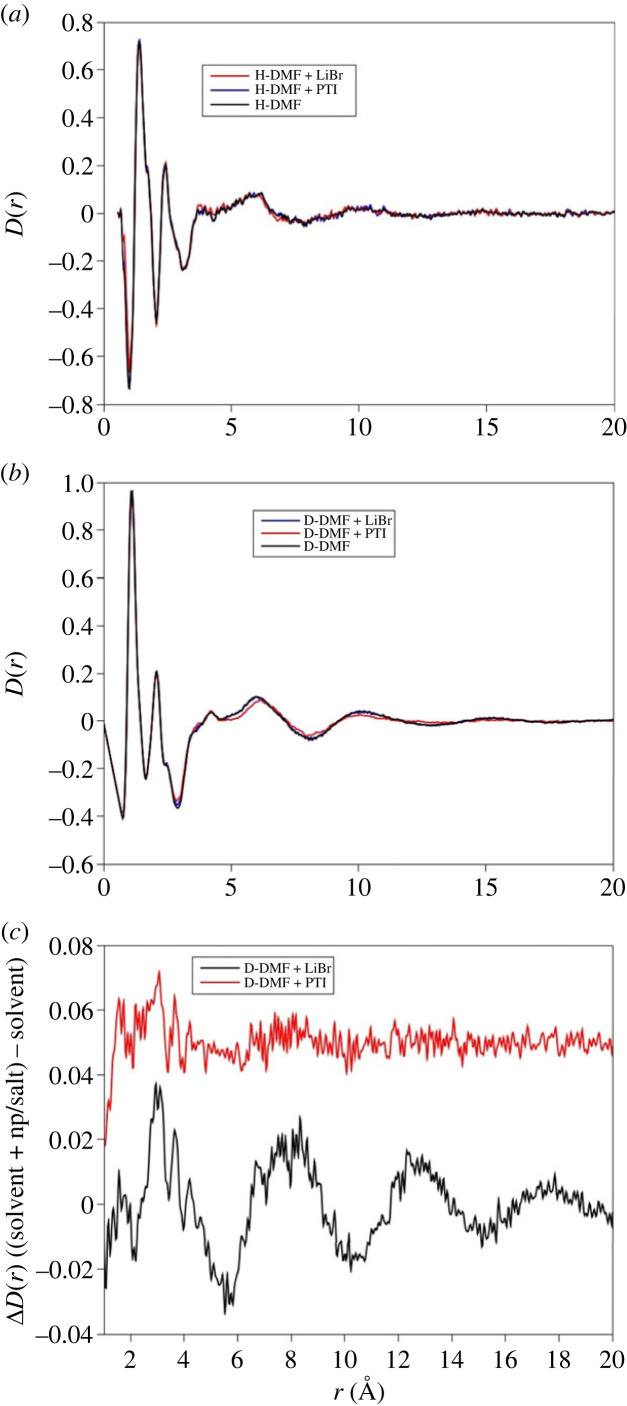
Real space neutron scattering data for DMF, DMF + PTI and DMF-LIBr for hydrogenated (*a*) and deuterated liquids (*b*). Also shown in the difference in *D*(*r*) between the solvent and the PTI solution and the saturated LiBr solution (*c*), the PTI differences are small and restricted to small radial distance while for the saturated solution correlations persist to 20 Å.

In contrast to the HEXRD data, the differences between the solvent and the solvent with PTI are restricted to differences in the first peak in the diffraction pattern (figure 4*b*). This contrasts with difference between the solvent saturated with LiBr and the solvent itself which are more distinct. There are minor differences in the real space transforms of the DMF and PTI solution (figure 5). For the saturated LiBr, the differences are more pronounced (figure 5*c*). These differences most likely represent structural changes in the H–H and C–H partial contributions associated with the solvation of Br^−^ and Li^+^ ions (table 2). In contrast to the HEXRD, the differences in the neutron scattering between the PTI solutions and DMF solvent are subtle, highlighting the differences in scattering between the two radiation types. For X-rays the scattering is from carbon atom pairs while for neutrons the scattering is mainly from hydrogen. A structural model refined against both neutron and X-ray data can therefore provide a detailed picture of solvent structural changes upon dissolving the PTI nanosheets.

### (c) EPSR modelling

**Figure 6.**
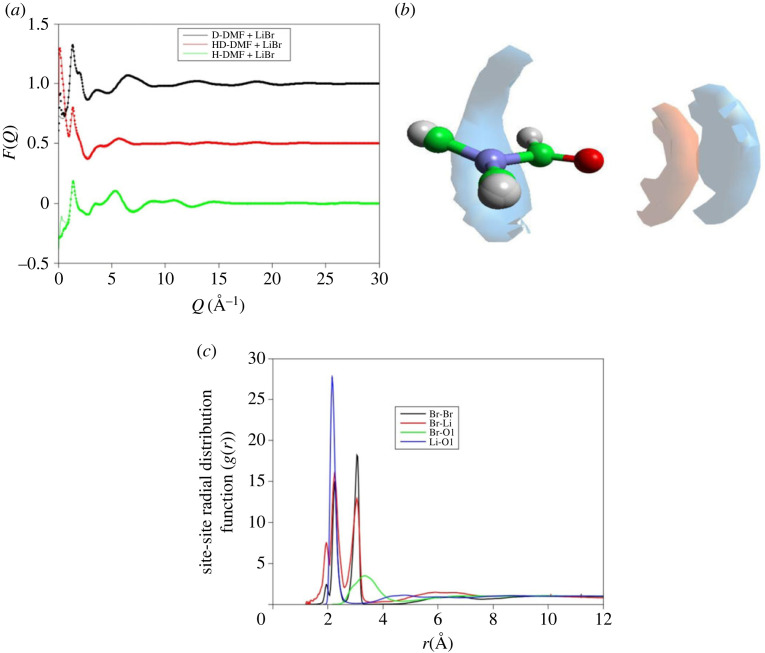
Results of EPSR fitting to saturated LiBr DMF (*a*), the spatial density function (SDF) for Br^−^ (blue) and Li^+^ (red) around an averaged DMF molecule (*b*) are shown defining a constrained Li–O and Li–Br distance, shown in the site–site partial contribution also obtained from the EPSR fits (*c*).

The structure of the saturated LiBr–DMF solution is compared with that of PTI solution initially. This is used to evaluate the contribution of the intercalating salt. On exfoliation of the PTI, the intercalation LiBr is dispersed into the DMF solvent and the apparent structural differences between the PTI solution and liquid DMF solvent could simply result from the structural changes resulting in the solvation of Br^−^ and Li^+^ by DMF molecules. Although the contribution of the Br and Li atom pairs to the total scattering are themselves small (table 2), the changes in the DMF structure would result in changes in (dominantly) the C–H and H–H contributions to the total scattering pattern. The isotopically tagged, LiBr-saturated DMF solutions were modelled using the EPSR model; here a configuration of 1000 DMF molecules was mixed with 85 Li^+^ and 85 Br^−^ ions. The density used was 0.0933 atoms Å^−3^ which yields a cubic box 51 Å^3^. The random configuration was allowed to equilibrate and form a stable configuration using the Lennard–Jones seed potentials (table 1) before the empirical potential then turned on and trajectory collected. The results after 30 000 iterations are shown in figure 6. The trajectory was used to determine the distribution of the Br^−^ and Li^+^ ions surrounding the DMF molecule, also shown in figure 6. An averaged DMF molecule with a dipole defined between the oxygen and nitrogen atoms [32] was used to develop the spatial density functions (figure 6*b*) for both Br^−^ and Li^+^. There are two bromine locations, one close to the formic and methyl hydrogens while the other, very closely aligned to the DMF oxygen, the lithium is tightly constrained between the Br− and oxygen. There is no solvent between the Li^+^ and Br^−^ ions and solvation of the LiBr is not too strong. This three-dimensional distribution of bromine and lithium is reflected in the site–site (figure 6*c*) partials for the saturated salt solution, the Li–Br partial resulting from EPSR is very sharp, the Li–O partial is similarly sharp while the broader Br–Br and Br–O correlations reflect a greater distribution of distances.

**Figure 7.**
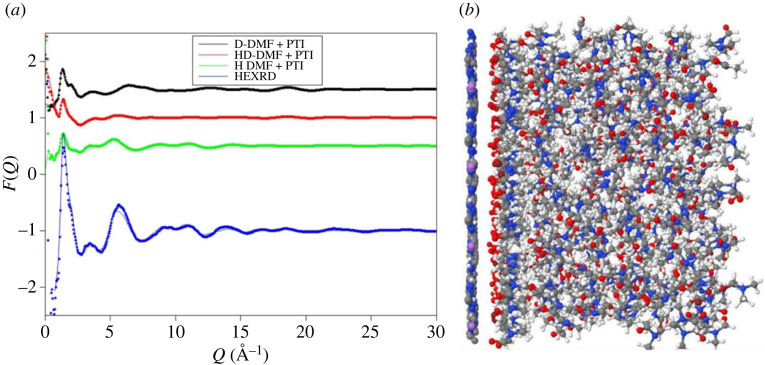
Results of the EPSR fit to the HEXRD and neutron scattering data, the latter with isotopic substitution (*a*). There diffraction data were collected on different samples, yet the simulation uses all four data sets to refine the model. The fits are remarkably good, there is a slight mismatch at approximately 6 Å in the X-ray pattern, which is possibly due to an incorrect normalization of the X-ray data. The snapshot of the simulation, also shown clearly shows the development of solvation shells at the PTI–DMF interface (*b*).

EPSR modelling was also used to determine the ordering of the solvent molecules around the PTI surface. It is important to state at this stage the assumptions made in the model. The gCN particles in the DMF liquid are two-dimensional hexagonal nanosheets which may be up to 100 nm (1000 Å) in diameter, but these are only a few, typically 9, layers thick. This means that the thickness of the nanosheets is around 2.5 nm (25 Å). The PTI does not contribute significantly to the total scattered signal, but the solvent structure is influenced by the presence of the nanoparticles and shows changes in structure up to 15 Å which although is comparable in one dimension is exceeded by the PTI in *a-* and *b*-directions. We use the bulk diffraction data to constrain the EPSR model which we then use to predict the interaction between the nanosheet surface and the solvent, the solvent in one direction. To do this a *single* layer of the PTI material was used. This was generated from the PTI crystal structure [21] extended in the *a-* and *b-*directions by 4-unit cells. The bromine ions were removed although the lithium ions are retained in the PTI layers. The *c*-axis direction is extended to 100 Å and the resulting space is filled with 1500 DMF molecules to match the solution density, charge-balancing Br^−^ is also added. The result is a single PTI layer immersed in 100 Å of DMF solution. We assume that the Br^−^ is released into the solvent when the PTI is delaminated and dissolved. We further assume no contribution of the PTI to the scattered signal and that the solvent-nanoparticle interaction can be constrained by the diffraction data even though the interaction at this interface is not directly measured, the structural change is not a result of Br-solvation and must reflect the PTI-solvent contribution. In the EPSR simulation, we aim to correlate the composition of the simulation with the experiment, and we chose a single layer rather than 9 layers to avoid using a very large simulation box. Although we considered this the best compromise, there is a further complication from using a single layer, since the EPSR model considers DMF molecules on both sides of the PTI sheet and so the solvent interactions are only modelled to half the box axis (i.e. 50 Å). Nevertheless, EPSR does accurately model the PTI monolayer in solution and can be used to at least suggest the changes in the DMF liquid structure when PTI is dissolved. The results of the EPSR fits for neutron and X-ray data are shown in figure 7 after 11 375 iterations.

The configuration that results from this modelling is also shown (figure 7*b*). This illustrates the predicted variation in density with distance from the PTI surface. The oxygen atoms within the DMF molecule are clearly orientated towards the PTI surface while there is a distinct band of carbon atoms, also parallel to the PTI surface.

**Figure 8.**
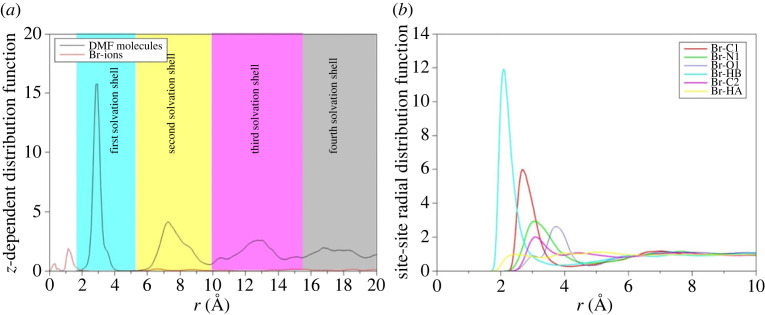
The *z*-dependent pair distribution function for DMF and bromine showing the ordering of the solvent and formation of at least four distinct solvation shells surrounding the PTI nanoparticles (*a*). There is also formation of a Br− layer close to the PTI surface. The site–site partial contributions for the Br-atom pairs (*b*) are also shown. This illustrates the coordination of the Br by the oxygen, carbon and nitrogen atoms in the DMF molecule, consistent with the HEXRD data.

The *z*-dependent pair distribution function is used to define the solvation shells surrounding the carbon nitride nanoparticle. Figure 8 shows the *z*-dependent variation in density of the DMF molecule with the solvation shells clearly defined by maxima in the pair distribution function. The distribution of Br^−^ is also shown and indicates that, as anticipated, the Br− ions are arranged close to the charged PTI surface although some are clearly present in the second solvation shell. The site–site partial distributions for Br^−^ to the DMF constituent atoms are also shown (figure 8*b*) which indicates that the Br is coordinated by the formic hydrogen on the DMF molecule with the coordination by the methyl groups relatively flat and explaining the relatively weak distinction between the neutron scattering data for the solvent and PTI solution. The correlations between the amidic carbon and both oxygen and nitrogen in the DMF molecule are consistent with the more obvious differences in the X-ray data.

## 4. Discussion

The neutron and X-ray data can be combined to constrain an EPSR model that provides a possible mechanism of dissolution of the PTI in DMF. When the intercalated PTI is dissolved in DMF, most, but not all, of the Br^−^ ions remain close to the PTI surface which is assumed to be charged because of the presence of Li^+^ and H^+^ in the PTI layer. In the EPSR model, the amidic carbon, oxygen and nitrogen from the DMF molecule are directed towards this layer of bromine and define distinctive solvation layers that persist to distances of at least 15 Å from the PTI-solvent interface. This is consistent with the formation of solvation shells shown in the real space transform of the measured X-ray total scattering data. Such strong solvent ordering can enable the energetically favourable configuration by lowering the enthalpy of the system and thus the free energy driving the observed spontaneous dissolution. There will be a related decrease in entropy although we note the DMF solvent itself is already relatively ordered [32], and thus the relative decrease is not expected to be relatively large. Although the presence of a layer of Br^−^ close to the PTI surface provides the charge balance necessary for the Li^+^ ions that remain in the PTI lattice it is possible that the solvent ordering may occur without this additional ion, and there are still questions that are not resolved. First, although PTI has been shown to dissolve in aprotic, polar solvents it does not dissolve in all of them [2]. The balance between the enthalpic and entropic contributions to the dissolution of PTI and degree of solvent ordering in different solvents may explain why dissolution occurs in some solvents but not in others. A similar situation occurs with graphene oxide, where dispersions or solutions are formed in some but not all polar solvents [35] including dispersions formed in DMF but not DMSO. This suggests that solvent ordering provides a mechanism for solution without necessarily involving the additional ions that are in exclusionary boundary layers. This underscores a second point: the synthesis of PTI involves a molten salt route with the halide ions intercalated within the graphitic carbon nitride structure; however, this salt can be removed (by Soxhlet extraction [19]) leaving an intercalant-free form which is also shown to be soluble in solvents such as DMF and DMSO, although the H^+^ exchanges with Li^+^ and the intercalant-free PTI is polar. Nonetheless, this study suggests that the ordering of solvent provides the energetic advantage required to form the PTI solutions.

## 5. Concluding remarks

Combined analysis of high-energy X-ray and neutron scattering data strongly indicate a change in the structure of the aprotic, polar solvent DMF when a solution of the graphitic carbon nitride, PTI is dissolved. This is most obvious in the high-energy X-ray diffraction pattern of the PTI solution with distinctive changes in the 4–6 Å^−1^ range and in real space the differences between solution and solvent persist to distances of up to 15 Å. These differences do not represent either the presence of a small amount of the PTI crystals, nor do they reflect the presence of Br− ions that are intercalated in the PTI and are dispersed on dissolution; the concentrations are too small to influence the scattering pattern in this way. Neutron scattering patterns for similar solutions show minor differences and are less distinct, most likely because the scattering is dominated by that from hydrogen in the DMF methyl groups whilst the X-ray scattering reflects scatter from carbon, oxygen and nitrogen. Further exploration of the data using EPSR analysis reveals the development of several solvation shells and a boundary layer of Br^−^ at the PTI-solvent interface. These data confirm the emerging evidence that indicates the breakdown of classical models of colloid formation in systems where the dimension of the dispersed or dissolved nanoparticles approaches the dimensions of the molecules that comprise the solvent. Although we cannot be certain whether the restructuring of the solvent around nanosheets is unique to this PTI–DMF liquid, we are currently undertaking further study using the same combined diffraction methodology on PTI nanosheets dissolved in DMSO as well as the ordering of water and DMF around two-dimensional materials including graphene oxide to establish the universality of these observations.

## Data Availability

Data and materials are available upon request from the authors.
